# Supplementary material for: New botulinum neurotoxin constructs for treatment of chronic pain
Source: Life Sci Alliance. 2023 Apr 11;6(6):e202201631. doi: 10.26508/lsa.202201631 (PMC10098373; doi:10.26508/lsa.202201631)
Supplement: Supplementary file 1 [file LSA-2022-01631_SdataFS2.pdf]

Figure S2A source data:

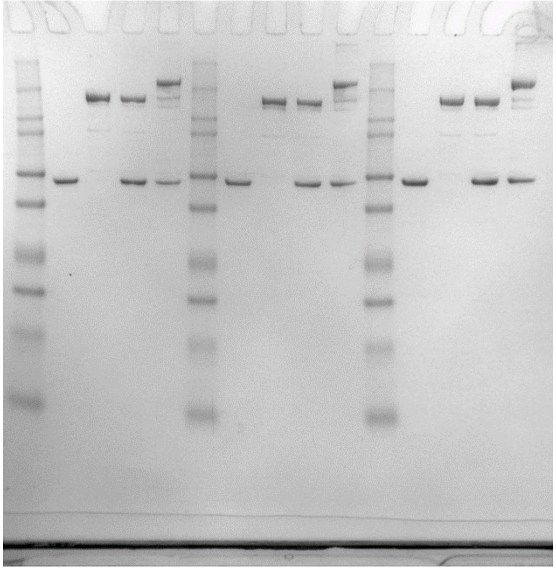

Lane 1	Protein Ladder (from top: 250 kDa, 150 kDa, 100 kDa, 75 kDa, 50 kDa, 37 kDa, 25 kDa, 20 kDa, 15 kDa, 10 kDa)
Lane 2	Spytag-Hc protein only
Lane 3	LHn-syx-Spy protein only
Lane 4	LHn-syx-Spy + Spytag-Hc at 0h (negative control)
Lane 5	LHn-syx-Spy + Spytag-Hc after 2h (el-iBoNT)
Lanes 6-15	Data irrelevant to this manuscript

Figure S2B Source data:

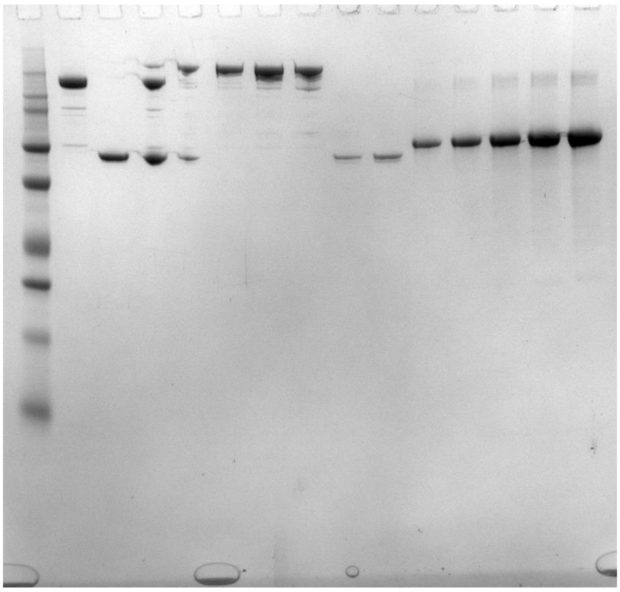

Lane 1	Protein Ladder (from top: 250 kDa, 150 kDa, 100 kDa, 75 kDa, 50 kDa, 37 kDa, 25 kDa, 20 kDa, 15 kDa, 10 kDa)
Lane 2	LHn-syx-Spy protein only
Lane 3	Spytag-Hc protein only
Lane 4	LHn-syx-Spy + Spytag-Hc at 0h (negative control)
Lane 5	LHn-syx-Spy + Spytag-Hc after 2h (el-iBoNT)
Lane 6	Fraction 8 from Size Exclusion Column
Lane 7	Fraction 9 from Size Exclusion Column
Lane 8	Fraction 10 from Size Exclusion Column
Lane 9	Fraction 18 from Size Exclusion Column
Lane 10	Fraction 19 from Size Exclusion Column
Lane 11	BSA Standard (0.4 ug)
Lane 12	BSA Standard (0.8 ug)
Lane 13	BSA Standard (1.2 ug)
Lane 14	BSA Standard (1.6 ug)
Lane 15	BSA Standard (2 ug)
